# Supplementary material for: Mitochondrial Targeting of Antioxidants Alters Pancreatic Acinar Cell Bioenergetics and Determines Cell Fate
Source: Int J Mol Sci. 2019 Apr 5;20(7):1700. doi: 10.3390/ijms20071700 (PMC6480340; doi:10.3390/ijms20071700)
Supplement: Supplementary file 1 [file ijms-20-01700-s001.pdf]

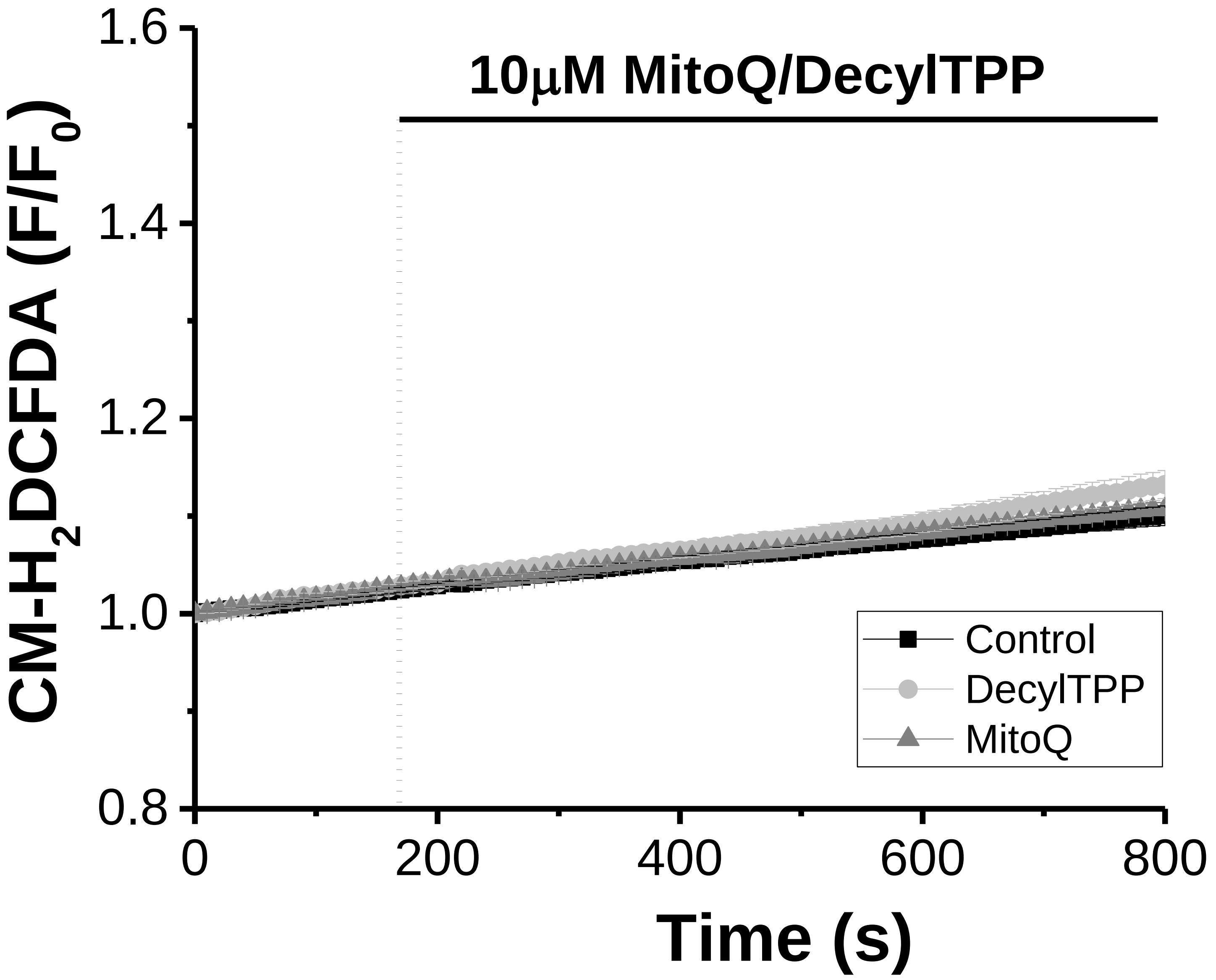

Supplementary Figure 1. Effects of MitoQ and DecylTPP on ROS in isolated pancreatic acinar cells measured using confocal microscopy. Neither 10 µM MitoQ nor DecylTPP increased ROS production compared to control ($n \geq 3$ mice per group). DCFDA fluorescence traces are shown as normalised (F/F_0) mean ± SEM.
